# Acoustic characterization for creep behaviors of marine sandy hydrate-bearing sediment

**DOI:** 10.1038/s41598-023-49523-1

**Published:** 2023-12-14

**Authors:** Yanlong Li, Qiaobo Hu, Nengyou Wu, Hongbin Wang, Xiaofeng Sun, Gaowei Hu, Zhiwen Sun, Yujing Jiang

**Affiliations:** 1 Key Laboratory of Gas Hydrate, Ministry of Natural Resources, Qingdao Institute of Marine Geology, Qingdao, 266237 China; 2 Laoshan Laboratory, Qingdao, 266237 China; 3 Sanya Offshore Oil and Gas Research Institute, Northeast Petroleum University, Sanya, 572025 China; 4 Graduate School of Engineering, Nagasaki University, Nagasaki, 852-8521 Japan

**Keywords:** Natural hazards, Environmental impact, Physical oceanography

## Abstract

Marine natural gas hydrate (NGH) is a promising substitutive low-carbon energy resource, whereas NGH-production induced geoengineering concerns remain challenging. Advanced forecast of possible geoengineering risks is the fundamental for eco-friendly NGH exploitation. Reservoir creep deformation is an early symptom of the geoengineering risks. However, whether the creep deformation behaviors of the NGH-bearing strata is predictable remains controversial. In this study, a series of multi-step loading creep test are conducted for sandy gas hydrate bearing sediment (GHBS) samples, during which the ultrasonic responses are recorded simultaneously. The acoustic velocity, compression-to-shear velocity ratio, Poission’s ratio, main frequency, and main frequency amplitude are used to characterize creep failures of the GHBS for the first time. Combining analyses of the creep behaviors and acoustic responses yield the following conclusions. Firstly, the long-term strength derived from creeping test is 0.45–0.60 times of the shear strength derived from triaxial shearing. Ignoring the creep effect might underestimate the scale and intensity of possible geoengineering risks during long-term NGH exploitation. Secondly, the acoustic velocity increases gently and then decreases continuously during creeping. Once the accelerated creep appears, the acoustic velocity plummets significantly, together with a sudden decrease in the compression-to-shear velocity ratio, and fluctuations in the main frequency and its amplitude. Furthermore, the main frequency and its amplitude shall fluctuate abruptly prior to the emergence of the accelerated creep. Therefore, we anticipate that the combination of abnormal fluctuations of main frequency and its amplitude can be used as early-warning indicators for possible creep failure of the GHBS. The results might have great significance for in-situ detection and prediction of possible reservoir failure during long-term NGH exploitation.

## Introduction

The global energy demands are increasing steadily with the ongoing prosperity and development of the human society, whereas the willingness for a cleaner living environments and the urgency of tackling climate change force the energy suppliers to upgrade the conventional energy chains^1,2^. However, the development of renewable energies (e.g. wind power, tidal power, solar power, hydrogen energy, biofuels) are currently far to regulate the immense contradictions between the energy supply uncertainties and the climate changing concerns, although they could act as good complements^3^. The fossil fuels shall remain the dominant role in the world’s energy supply chain in the coming decades^4^, among which natural gas is the most promising substitutes to realize a diversified, decarbonized energy supply chain.

Natural gas hydrate (NGH), as one of the primary sources of natural gas, is a kind of caged compound formed from water and natural gas (mostly methane in nature) under relatively high-pressure and low-temperature^2,5^. NGH is widely distributed in nature, mainly in the permafrost regions (< 10%) and subsea sediment in the continental margins (≥ 90%) such as the South China Sea^6^, Greenland shelf margin^7^, Krishna–Godavari basin^8^, eastern part of the Korean Peninsula^9^, and Cascadia margin^10^. It is anticipated that NGH accounts for thousands of trillions of cubic feet of methane gas, although the specific estimated amount range over several orders of magnitude^11^ and the recoverable amount remains even uncertain. With the proposal of the zero net-carbon emission ambitions, the NGH is widely accepted as a promising alternative energy resource by the Asian-Pacific countries such as China, Japan, India, and South Korea^11,12^. At the same time, the interactions between NGH and global climate change also attracts tremendous attentions^13–15^, since the NGH is undoubtedly the most important carbon sink on this planet and the change in NGH system would inevitably influence the Earth’s carbon cycle.

Therefore, exploiting the marine NGH in an eco-friendly way is the emerging demand to compromise the contradictions between fossil fuel supply and global changes^16^. However, great challenges such as poor reservoir characterization, immature exploration and exploitation techniques, possible geoengineering risks and/or disasters^17,18^ pose significant uncertainties for the safe and effective exploitation of NGH. Among these challenges, the geoengineering risks are highly related to the deformation characteristics of the gas hydrate-bearing strata (GHBS)^17,19^.

Geoengineering risks related to marine NGH exploitation includes but are not limited to sand production^20,21^, reservoir instability^22^, seafloor subsidence^23,24^, uncontrollable CH_4_ emission^25^, and possible slope failure^26,27^. All these geological risks are anticipated to be related to the time-dependent plastic deformation (i.e. creep) of the non-diagenetic marine GHBS^28,29^, since the NGH dissociation would undoubtedly deteriorate the strength^30^ and change the pore structures^31^ of the GHBS. However, quantitative relationships between creep characteristics and geoengineering risks during NGH exploitation remain unclear to some extent^32^.

Geoengineering-based techniques such as pressuremeter test^33^ and flat dilatometer test (DMT)^34^ could obtain failure behaviors of the GHBS directly. However, these emerging techniques are limited to laboratory experimental verifications to date. The triaxial shearing test plays a leading role in the creep test of GHBS from the laboratory experimental perspectives^35^. Based on the triaxial shearing test, Miyazaki et al.^36^ found that the creep of GHBS is much larger than that of water-saturated sand that is free of hydrate. Li et al.^37^ stated that the GHBS would be damaged rather than remain in the stable-creep stage when the deviator stress exceeds the quasi-static strength. Afterwards, Nakashima et al.^38^ pointed out that the creep behaviors of GHBS change depending on the loading method and stress rate. Zhou et al.^39^ distinguished the time-dependent deformation caused by effective stress rise from that induced by NGH saturation loss. Most recently, our experiments observed three typical creep stages of deceleration creep, stable creep, and accelerated creep for GHBS by deploying the multi-step loading method^40^. These findings provide significant guidance to understand the mesoscopic creep behaviors of GHBS, whereas the possibility of field monitoring for creep of GHBS remains unknown.

Compared with the geoengineering-based techniques, geophysical-based methods could obtain the reservoir information in a non-invasive, wide-coverage, and field-applicable way^41,42^. There were a few attempts to characterize the deformation behaviors of the marine GHBS via the onboard geophysical-based methods. For example, a large-scale flow-like deformation within the GHBS at the east coast of New Zealand was identified with the utilization of high-resolution multichannel seismic refection (MSR)^28^. In 2017–2018, the International Ocean Discovery Program (IODP) Expedition 372 used the logging while drilling (LWD) technique to identify relationships between NGH and sediment creep deformation^43,44^. These trials enlighten us that the acoustic-based geophysical techniques (e.g. MSR, LWD) could be of significant help in field monitoring of GHBS creep, whereas careful indoor feasibility test are required before field application.

Bridging the geophysical-based observation and geomechanical-based detection method is essential to fully characterize the time-dependent deformation of GHBS, to accurately formulate a generally applicable constitutive model, and to narrow the gaps between laboratory results and field applications. Therefore, we developed a device to test time-dependent deformation and the ultrasonic response of the GHBS under high pressure and low temperature. This paper reports the first set of integrating test results for sandy GHBS under multi-step loading conditions. Creep characterization of the GHBS are comprehensively conducted based on the relationships between acoustic signals and deformation data. The results shall provide references for accurate prediction of GHBS deformation and reservoir creep monitoring during long-term NGH exploitation.

## Experiments

### Experimental device

**Figure 1:**
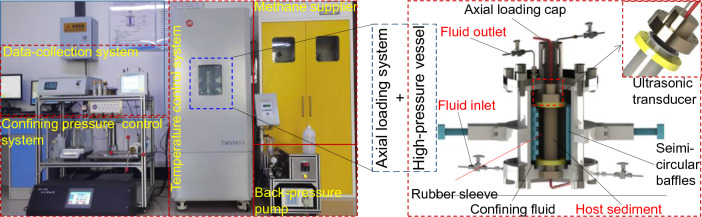
Photograph of the experimental device (left) and 3D diagrams of the high-pressure vessel (right). The system is composed of a data-collection system, a confining pressure control system, a step-in temperature control system, a methane supplier, an axial loading system, and a high-pressure vessel. The high-pressure vessel is the main space for hydrate-bearing sample preparation and creep test. The time-stress–strain curves are obtained by the axial loading module. Additional ultrasonic transducers are equipped at each side of the sample in the height-direction to measure acoustic signals during hydrate formation and creep test.

The diagram and photo of the experimental device are shown in Fig. 1. A detailed description of the device can be found in our previous publication^45^. The high-pressure vessel is able to synthesize artificial GHBS samples with a height of 120 mm and a diameter of 39 mm. The high-pressure vessel is able to withstand a pressure up to 30.0 MPa. During creep test, the axial loading control system can apply constant load on the sample, with a control accuracy of ± 0.002 kN. Axial strain is detected by the axial-loading sensor with a displacement accuracy of ± 0.001 mm. The volumetric strain is measured by the changes in the amount of confining-pressure liquid.

The ultrasonic transducers equipped at both ends of the GHBS sample record both shear wave (S-wave) and compression wave (P-wave) during loading. This process is realized by the combination of a Twave40612 high-frequency arbitrary waveform generator, an AG1016 power amplifier, an EVOC industrial computer, an ultrasonic transducer, an ultrasonic channel switching device, an oscilloscope, and a supporting software program. It is noted that ultrasonic signals filtering and denoising^46,47^ are required before the original data is used to characterize creep behaviors of GHBS.

As a result, both the geomechanical-based data (i.e. time-stress–strain) and the geophysical-based data (i.e. acoustic signals) could be obtained during the creep test for GHBS. The former is a direct indicator of creep behaviors and can be used to derive macroscopic creep parameters such as long-term strength. The later can be used as an appendix to explain possible creep failure mechanisms. Combination of these data enables us to model geophysical responses of creep behaviors for the reservoir during long-term NGH exploitation.

### Experimental procedures

In this study, sandy sediment with a grain-size range of 0.15–1.0 mm is used as host sediment for artificial methane hydrate. The average porosity of the sediment is 38.8%. Methane with a purity of 99.9% and deionized water are used to synthesize hydrate.

The sample preparation initiates by mixing a certain amount of pure water and sediment. The mixture is transferred into the rubber sleeve and pressed into a column sample within the high-pressure vessel. The step is followed by injecting methane into the sediment and increasing the confining pressure simultaneously. This process is continued until the pore pressure reaches 7.0 MPa. Afterwards, the pressurized vessel is kept standstill for around six hours at ambient temperature for possible leakage check. Then, the temperature control system is switched on and set as 1.0 ℃ to cool the vessel. It is noted that the effective confining pressure should be maintained constant (i.e. 0.5 MPa) during the aforementioned pressurization and cooling processes. Methane hydrate shall be synthesized during cooling, leading to pore-pressure decrease. Once the pore-pressure remains stable for more than six hours, it is assumed that all water molecules inside the sediment are crystallized into hydrate, indicating the finalization of sample preparation. The average hydrate saturation (*S*_h_) is controlled by the amount of water added into the sediment, and it is set to be 20%, 30%, and 40%, respectively, in this study.

The pore pressure is adjusted to be 6.1 MPa when the sample preparation is finished, whereas the effective confining pressure is set to be 1, 2, and 3 MPa, respectively. The multi-step loading method is applied in the experiments. Take the samples with an average hydrate saturation of 30% as an example, the specific loading paths for the creep tests are listed in Table 1. To laterally compare the creep failure characteristics under different confining pressure, the loading value is normalized into stress level in Table 1. The stress level is defined to be the ratio between the current load and the static shear strength of the sample obtained from triaxial shearing. According to Miyazaki et al.^48^, the static strength for sandy methane-hydrate bearing sediment (*S*_h_ = 30%) is 4.50, 7.93, 9.53 MPa, when the effective confining pressure is 1, 2, 3 MPa, respectively.

**Table 1:** Creep test loading path for sandy hydrate-bearing sediment when the average hydrate saturation is 30%.

Loading path	1 MPa		2 MPa		3 MPa	
	Load /MPa	Stress level	Load /MPa	Stress level	Load /MPa	Stress level
Level 1	2.4	0.53	3.0	0.38	2.7	0.28
Level 2	2.6	0.58	3.4	0.43	3.3	0.35
Level 3	2.8	0.62	3.8	0.48	3.9	0.41
Level 4	3.0	0.67	4.0	0.51	4.2	0.44
Level 5	–	–	4.3	0.54	4.5	0.47
Level 6	–	–	4.5	0.57	4.8	0.50

Firstly, a relatively low axial load (i.e. *level 1*) is applied to the GHBS sample. Then both the strain and the acoustic signals are recorded continuously. A higher axial load (i.e. *level 2*) is applied when the transient deformation rate is decreased below 0.003 mm/h. The duration for each loading level lasts for 24 h at least and 5 days at most, which is determined by the transient deformation rate. The creep test is finished when an accelerated creep stage is observed.

## Creep behaviors

### Strain curves

**Figure 2:**
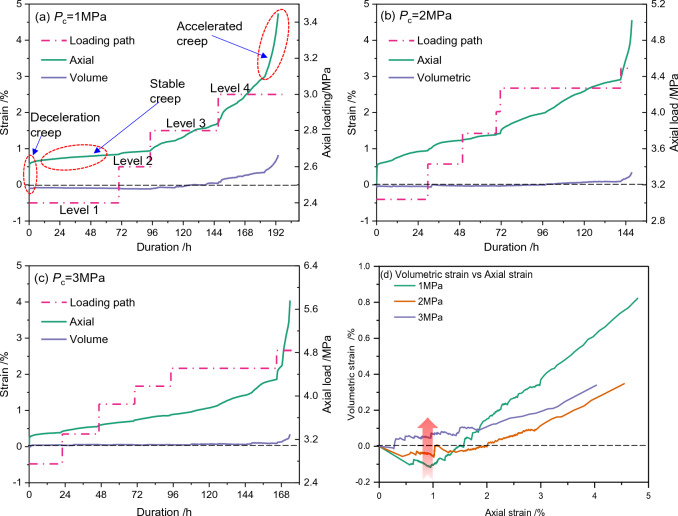
The evolution behaviors of axial strain, volumetric strain during creeping for sandy GHBS and the corresponding loading paths (dash-dotted line) when the average hydrate saturation is 30%. The relationship between volumetric strain and axial strain in (**d**) is extracted from (**a**) to (**c**).

Figure 2 shows the measured axial strain and volumetric strain under different loading paths (see Table 1) when average hydrate saturation is 30%. The loading paths are presented as pink dot-dash lines in Fig. 2a–c. Three different deformation stages of deceleration creep, stable creep, and accelerated creep can be identified from the axial strain curves. The deceleration creep and the stable occur successively at each of the loading level. The duration of deceleration creep is much shorter than that of the stable creep. However, the accelerated creep, which is a symbol of complete sample failure, appears only at the last loading level.

Negative volumetric strain values are observed in Fig. 2d, indicating an obvious shrinkage of the GHBS samples at the early stages of deformation. The maximum volumetric shrinkage reaches 0.12% when the effective confining pressure is 1.0 MPa (Fig. 2a). Such a shrinkage trend is suppressed by increasing effective confining pressure, and there are no obvious negative value for volumetric strain when the effective confining pressure is increased to 3.0 MPa (Fig. 2d). Under the same effective confining pressure (e.g. 1.0 MPa), the volumetric strain transfers into positive value and increases nonlinearly at the middle-later stages of loading, indicating dilation of the sample. The shrinkage of the volumetric strain complies with the results obtained from triaxial shearing^30,49^, and shall be attributed to the changes in the internal pore space of the sample.

### Long-term strength

In the multi-step loading creep test, the axial loading at which the accelerated creep appears is 3.0, 4.5, and 4.8 MPa, respectively, when the effective confining pressure is 1, 2, and 3 MPa. This implies that the creep failure of GHBS would somehow mitigated with the increase of confining pressure. Such effects can be quantitatively evaluated by the long-term strength (*S*_c_), which is defined as the maximum axial load under which the sample is able to remain stable during long-term loading^50,51^. Here, the transitional creep method^52^ is introduced to predict the long-term strength. The ratio of the shear strength to the long-term strength can be defined as the normalized failure stress.

**Figure 3:**
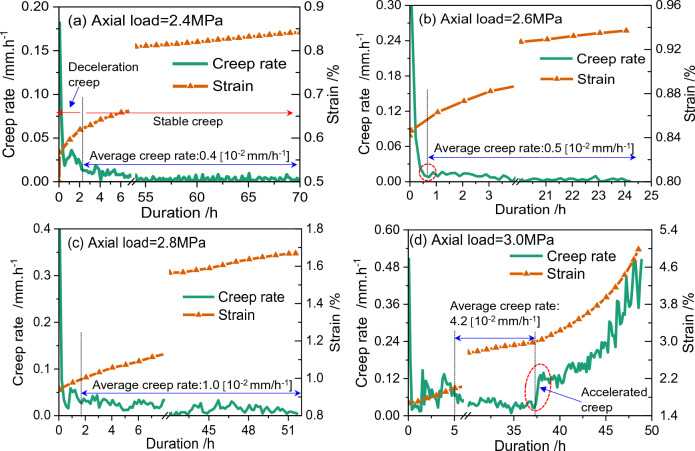
Transitional creep rate and cumulative axial strain evolutionary behaviors for GHBS when effective confining pressure is 1 MPa and average hydrate saturation is 30%. When the axial load is increased from 2.8 to 3.0 MPa, the shape of the transitional creep rate curves changes from an *L*-shape into a *U*-shape. The long-term strength is supposed to locate in the range between 2.8 and 3.0 MPa.

**Figure 4:**
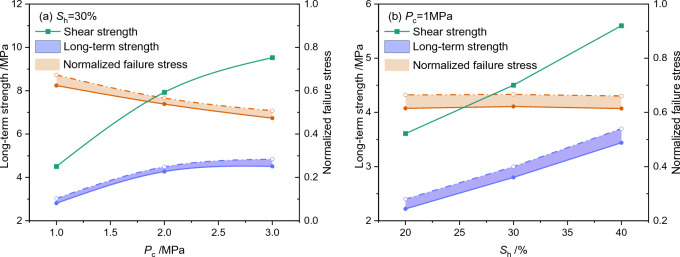
Influence of effective confining pressure (**a**) and hydrate saturation (**b**) on the shear strength obtained from triaxial shearing, long-term strength, and the normalized failure stress under creeping. The shear strength is cited from Miyazaki et al.^48^. The normalized failure stress is the ratio of the shear strength to the average of long-term strength.

Creep rate is another symbol to characterize the creep behaviors of GHBS. An example of the transitional creep rate evolution behaviors for sandy GHBS is shown in Fig. 3. The data in Fig. 3 is derived from Fig. 2a, when average hydrate saturation is 30% and effective confining pressure is 1 MPa. It could be seen from Fig. 3a–c that the creep rate decreases rapidly during the deceleration creep stage, and then decrease moderately during the stable creep stage. The transitional creep rate curves exhibit an “*L*” shape when the axial load is less than the long-term strength. On the other hand, it could be seen from Fig. 3d that the transitional creep rate becomes a “*U*” shape, once the axial load exceeds the long-term strength. Hence, such a *U*-shaped creep rate curve is a symbol of sample failure. As a result, we define the critical axial load at which the transitional creep rate curves transfers from *L*-shape into *U*-shape as the long-term strength. It could be concluded from Fig. 3 that the long-term strength (represented as *S*_c_) for GHBS (*S*_h_ = 30%) should be 2.8 MPa ≤ *S*_c_ < 3.0 MPa, when the effective confining pressure is 1 MPa. The long-term strength predicted for the other experiments are depicted in Fig. 4.

A comparison among the long-term strength, the failure stress level, and the shear strength is depicted in Fig. 4. It could be seen that both the shear strength and the long-term strength decay with the decrease in either effective confining pressure or average hydrate saturation. The long-term strength ranges 0.45–0.60 times of the shear strength under the same effective confining pressure and the same hydrate saturation. This enlightens the importance of taking the long-term creep effect into consideration during geoengineering risks estimation. The conventional risk-evaluation method, which ignores the creep effect but solely depends on shear strength, might underestimate the scale and intensity of possible geoengineering risks during long-term NGH exploitation.

Besides, the normalized failure stress decreases with the increase in effective confining pressure, enlarging the gap between the shear strength and the long-term strength (Fig. 4a). However, the influence of hydrate saturation on the normalized failure stress seems to be negligible (Fig. 4b). This indicates that hydrate saturation might affect the failure mechanisms of GHBS under long-term loading (i.e. creep) and rapid shear (i.e. triaxial shear) equivalently. It is anticipated that the hydrate saturation influences the shear strength by changing either the bulk density^53^ or hydrate morphologies^54^ from the microscopic perspectives.

However, the influencing mechanisms of effective confining pressure on creep and triaxial shear vary significantly (Fig. 4b). The confining pressure mainly affects the shear strength by enhancing the interlocking forces among particles^55^. However, the long-term loading might induce minor phase changes at the contacting interfaces of different particles^31,56^. Then a thin fluid layer might be formed at the contacting interfaces, although the amount of pressure-melting NGH is negligible regarding saturation change. The fluid layer might act as a lubricant for the relative movement of particles, alleviating the interlocking forces among particles.

## Acoustic characterization

### Acoustic velocity

**Figure 5:**
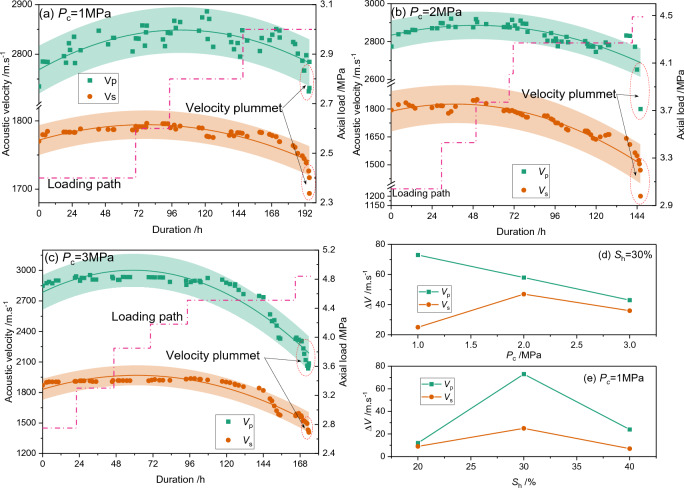
S-wave and P-wave velocity changing behaviors during creeping. The hydrate saturation in (**a**)–(**c**) is 30%. *V*_s_ represents shear-wave velocity, and *V*_p_ represents compression-wave velocity, respectively. The *ΔV* is difference between the maximum velocity and its initial value. The shadow areas in (**a**)–(**c**) represent confidence intervals of 90%.

The acoustic signals are acquired throughout the hydrate formation process and the creep test. The acoustic responses during hydrate formation have been well-stated in the previous publications (e.g. references^46,47^), and the acoustic signals remain constant with time when hydrate formation is finished in this study. During creep test, both the compression wave (P-wave) and the shear wave (S-wave) are acquired and the head wave of the acoustic signals are extracted. The P-wave and S-wave velocities for GHBS with an average hydrate saturation of 30% are shown in Fig. 5a–c. It could be seen that the velocities of both the P-wave and S-wave witnessed a sudden plummet at the end of loading. The plummet occurs simultaneously with the occurrence of accelerated creep (see Fig. 3). Therefore, the plummet of acoustic velocity can be used as a prominent identification for creep failure of GHBS.

Besides, the velocity curves increase gently at the early stages of creep and then decrease continuously at the middle-later stage. Hence, a maximum velocity value exists on the velocity–time curves. Generally speaking, the increase of acoustic velocity is induced by structural compression, whereas the decrease of acoustic velocity represents dilation for a single-phase homogeneous medium. However, a comparison between Fig. 5 and Fig. 2d implies that the changing point of volumetric strain from negative into positive value appears much earlier than the appearance of the maximum velocity value. This implies that the structural changes within the HBS are much more complex than the single-phase homogeneous medium. There is no doubt that the GHBS is a multi-phase inhomogeneous medium. The evolutionary behaviors of the acoustic velocities for such a multi-phase inhomogeneous medium might be a combing result of the aforementioned pressure melting, contacting interface changes between different phases, and failure mode (i.e. compaction or dilation).

The difference between the aforementioned maximum acoustic velocity and its initial value is used to characterize the acoustic response sensitivity during creeping. The velocity differences under different effective confining pressure and different hydrate saturation are shown in Fig. 5d, e. It could be concluded from the Figures that the P-velocity is more sensitive to the creep deformation than S-velocity. The P-velocity difference decreases linearly with the increase of effective confining pressure, whereas the S-velocity difference increases and then decreases with the increase of confining pressure.

### Compression-to-shear velocity ratio

**Figure 6:**
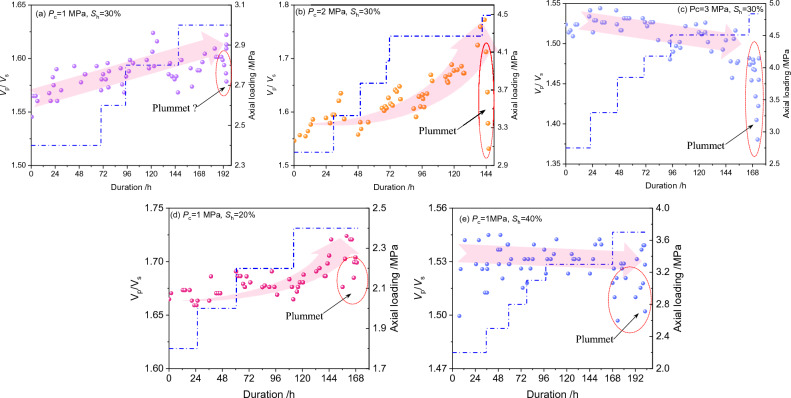
Evolutionary behaviors of compression-to-shear velocity ratio (*V*_p_/*V*_s_) during creeping. The creep strain curves related to (*d*) and (*e*) can be found in reference^40^ and were not included here. The blue dash-dotted line represents the loading path during multi-step loading. The flash scattering points represent the predicted* V*_p_/*V*_s_ value.

The compression-to-shear velocity ratio (*V*_p_/*V*_s_) is widely used to characterize the GHBS in the geophysical communities. The *V*_p_/*V*_s_ during creep loading are shown in Fig. 6. It could be seen that the *V*_p_/*V*_s_ would witness a sudden plummet once the accelerated creep appears, although the reduction degree differs for different experiments. It seems safe to conclude that the sudden plummet of the* V*_p_/*V*_s_ can be used as a prominent identification for creep failure of GHBS. The reduction degree of the* V*_p_/*V*_s_ is speculated to be related to the creep failure intensity or fracture mode. The joint application of digital-based technologies (e.g. X-CT) is required to uncover the relationships between creep failure mode and acoustic responses in future work.

The uncertainties in the *V*_p_/*V*_s_ evolutionary behavior lie in the time intervals prior to the occurrence of accelerated creep. In Figs. 6a (i.e. *P*_c_ = 1 MPa, *S*_h_ = 30%), 6b (i.e. *P*_c_ = 2 MPa, *S*_h_ = 30%), and 6d (i.e. *P*_c_ = 1 MPa, *S*_h_ = 20%), the *V*_p_/*V*_s_ increases gradually either in a linear mode or power-functional mode during the time intervals prior to the occurrence of accelerated creep. However, a slight decreasing trend in the *V*_p_/*V*_s_ is observed in Figs. 6c (i.e. *P*_c_ = 3 MPa, *S*_h_ = 30%), 6e (i.e. *P*_c_ = 1 MPa, *S*_h_ = 40%). We could primarily anticipate that the *V*_p_/*V*_s_ increasing trend occurs under relatively low hydrate saturation and/or low confining pressure conditions, whereas the *V*_p_/*V*_s_ decreasing trend occurs under relatively high hydrate saturation and high confining pressure conditions.

Generally speaking, the plummet in the *V*_p_/*V*_s_ indicates the occurrence of creep failure, whereas the gradual changing of the *V*_p_/*V*_s_ in the early stages can be viewed as a symbol of pore structure regulation. Compared with the S-wave velocity, it has been proven that the micro pore-structure changes are sensitive to P-wave velocity^57^. This could be also inferred from the aforementioned Fig. 5d, e. The *V*_p_/*V*_s_ decreases once the pore structure is compacted, and vice versa. Under certain axial load, the shrinkage of the pore structure shall be hindered by either high hydrate saturation or confining pressure. This might be the inducement of the minor decreasing trend in Fig. 6c and Fig. 6e.

### Poisson's ratio

The acoustic signals are important identifications for elastic parameters of the GHBS, such as dynamic Poisson’s ratio and dynamic Young's modulus. However, these acoustic-based elastic parameter prediction methods are based on the assumption that the reservoir remains in a steady state. The Poisson’s ratio can be predicted via Eq. (1), which is a function of the P-to-S velocity ratio^58^. If the GHBS is damaged under long-term loading, the predicted results shall become anomalous or even become negative. Under these circumstances, the calculation results from Eq. (1) cannot be used to characterize the properties of the GHBS. However, the sample changing processes from steady state into failure state might be reflected by the changes in Poisson’s ratio. Therefore, we still use the Poisson’s ratio here, trying to figure out an identification for creep failure of GHBS.

1 $$\mu = \frac{{\frac{1}{2}\left( {V_{p}^{{}} /V_{s}^{{}} } \right)^{2} - 1}}{{\left( {V_{p}^{{}} /V_{s}^{{}} } \right)^{2} - 1}}$$

where, *V*_p_ is the P-wave velocity, m/s. *V*_s_ is the S-wave velocity, m/s.

**Figure 7:**
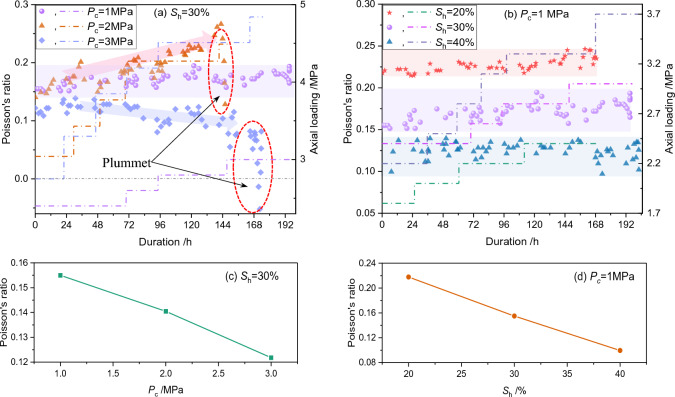
Poisson's ratio evolutionary behaviors during loading (**a**, **b**) and the Poisson’s ratio at the initial state (**c**, **d**).

The change behaviors of Poisson’s ratio under different effective confining pressure and hydrate saturation are displayed in Fig. 7. Under the initial state (i.e., when the time duration is 0 h), the Poisson’s ratio decrease with the increase in either hydrate saturation or confining pressure (see Fig. 7c, d). This is consistent with the previous findings from both the geophysical interpretation^59^ and triaxial shearing test^30,48,60^, which confirms the reliability of the current experiments.

Besides, the Poisson's ratio of the GHBS remains stable (i.e., ranges randomly within an acceptable narrow range) throughout the loading processes under all hydrate saturation conditions when the confining pressure is 1.0 MPa (see Fig. 7b). This indicates that the Poisson’s ratio is unable to reflect the creep failure processes under such conditions. However, the Poisson's ratio shows abrupt decrease once the accelerated creep stage appears under relatively high confining pressure (see Fig. 7a,* P*_c_ = 2 MPa, 3 MPa). The predicted Poisson’s ratio even drops to negative when confining pressure is 3 MPa. A more interesting phenomenon is that the Poisson’s ratio increases continuously during the loading interval prior to the occurrence of accelerated creep when *P*_c_ = 2 MPa. However, the changing tendency comes in the opposite direction when *P*_c_ = 3 MPa. This confuses us in the current stage, since we are unable to give a confident answer and further comprehensive studies are needed to clarify mechanisms.

### Main frequency

**Figure 8:**
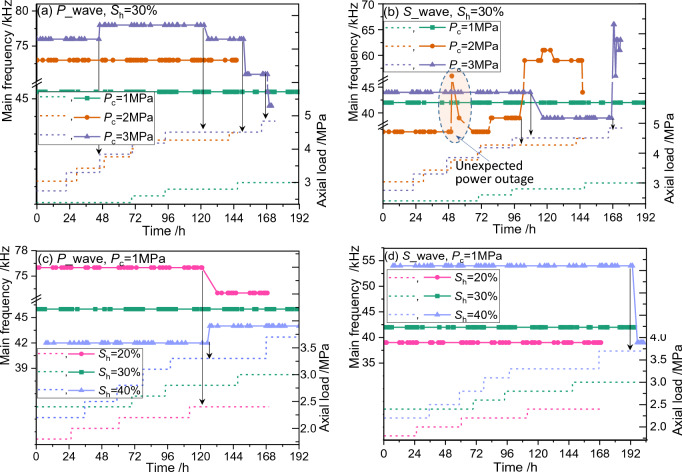
A comparison of loading path and acoustic main frequency for sandy hydrate-bearing sediment during creeping. The loading paths are depicted in dot line, and the main frequency is presented in marked solid line. (**a**) and (**c**) Compare the evolutionary behaviors of the main frequency of the compressible wave under different experimental conditions. (**b**) and (**d**) Compare the evolutionary behaviors of the main frequency of the shear wave under different experimental conditions.

Both the changes in the main frequency and its amplitude may imply structural changes within the sample, although relationships between these parameters and geotechnical properties were rarely reported and the response mechanisms remain unclear. The acoustic-source main frequency of the Twave40612 high-frequency arbitrary waveform generator is 50 kHz. The main frequency and its amplitude for the GHBS during creeping can be obtained through Fourier transformation^61^ of the received acoustic waves. Main frequencies of both the P-wave and S-wave during long-term loading are shown in Fig. 8. It is noted that the abnormal fluctuation in Fig. 8b around *t* = 50 h (*S*_h_ = 30% and *P*_c_ = 2 MPa) is a result of an unexpected power outage in the lab. The main frequency returns to a normal state within a short interval once the power is plugged in, and thus, the influence of power outage is negligible.

It could be concluded from Fig. 8 that the changes in the main frequency usually occur in the blink of an eye, without obvious transitional duration. When such a change appears, we cannot observe any noticeable changes in the axial strain curves (see Fig. 3). Such distinct differences in geophysical signals (i.e. acoustic main frequency amplitude) and geoengineering responses (i.e. axial strain) give us significant insights to predict deformation behaviors of the GHBS from diverse perspectives. The abrupt changes in acoustic signals might be ascribed to the micro-structural changes of the GHBS, which are unable to be detected by the strain gauges. Furthermore, most of the changes in the main frequency occur at the state when the axial load approaches or exceeds the long-term strength (see Fig. 4) of the sample. Namely, the sudden changes in the main frequency usually occur prior to the appearance of accelerated creep stage (see Fig. 2). This is quite crucial as it implies that the main frequency would somehow be used as an early-identification for creep failure of the GHBS.

There are two very puzzling phenomena in Fig. 8. Firstly, the changes in the P-wave main frequency usually don’t occur simultaneously with the changes in the S-wave main frequency. The P-wave main frequency remains constant when the S-wave main frequency changes, and vice versa. Theoretically, it is possible that different types of structural changes or failure fractures would appear in the GHBS during creeping. The aforementioned phenomenon proves that the sensitivity of main frequencies of P-wave and S-wave are different for different micro-structural failure modes. Secondly, whether the main frequency increases or decreases seems to occur randomly. This is another evidence that the creep shall induce different micro-structural failure modes within the GHBS.

However, the main frequency remains constant throughout the creep test under some special experimental conditions (e.g. *S*_h_ = 30% and *P*_c_ = 1 MPa, marked in blue color in Fig. 8). This seems to indicate that there are some unknown factors remaine exposed, which suppresses the main frequency changes induced from micro-structure changes for GHBS. This causes trouble for creep failure prediction and characterization. Therefore, additional criteria, such as main frequency amplitude, are needed for these cases. The following section would focus on the evolutionary behaviors of the main frequency amplitude for those cases when the main frequency remains unchanged.

### Main frequency amplitude

**Figure 9:**
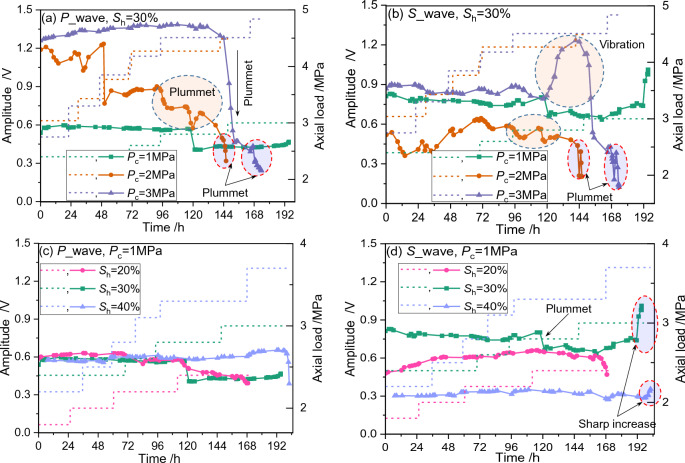
A comparison of loading path and acoustic main frequency amplitude for sandy hydrate-bearing sediment during creeping. The loading paths are depicted in dot line, and the main frequency is presented in marked solid line. (**a**) and (**c**) Compare the evolutionary behaviors of the main frequency amplitude of the compressible wave under different experimental conditions. (**b**) and (**d**) Compare the evolutionary behaviors of the main frequency amplitude of the shear wave under different experimental conditions. Typical changes marked with red-edged circles occur when the axial load reaches the long-term strength, whereas that marked with light blue-edged circles occur at the moment when accelerated creep appears.

The main frequency corresponding to the aforementioned main frequency is shown in Fig. 9. It could be seen that the main frequency amplitude could rarely keep constant throughout the creep test, implying that the micro-structures change and regulate dynamically during the long-term loading. The increase in the confining pressure seems to amplify the fluctuations of the main frequency amplitude (Fig. 9a and b), whereas the changes in the hydrate saturation have little influence on the fluctuation amplitude of the main frequency amplitude (Fig. 9c and d). It is anticipated that the main frequency amplitude is more sensitive to the increase of confining pressure (equivalent to pressure drop) than the decrease of hydrate saturation (equivalent to hydrate dissociation) during field NGH exploitation.

There are two abrupt changes in each of the main frequency amplitude curves during long-term loading. The first abrupt change appears when the axial load reaches the long-term strength. The abnormal fluctuation of the main frequency amplitude appears prior to the occurrence of the accelerated creep, and this abnormal fluctuation can also be used as an effective symbol for early forecast of creep failure. The second abrupt change occurs when the accelerated creep appears. At this point, the P-wave main frequency amplitude plummets, except for the case when *S*_h_ = 30% and *P*_c_ = 1.0 MPa (Fig. 9a). At the same time, the S-wave main frequency amplitude plummets or increases sharply.

As a result, the combination of the sudden changes in the P-wave and S-wave main frequency amplitudes could be used as an early forecast symbol of reservoir creep failure during NGH exploitation. However, it is worthy of noting that the aforementioned general fluctuations of the amplitude might be caused by either the changes of main frequency or the structure changes of the GHBS. Only the fluctuations induced from structure changes could be used as a secondary indicator for creep deformation. In another word, the aforementioned abrupt changes of the main frequency is the first indicator for advanced forecast of creep failure of the GHBS. The main frequency amplitude could be used as the secondary indicator for advanced forecast of creep failure of the GHBS only if the main frequency remains stable.

### Discussions and perspectives

During the long-term loading, the changes in acoustic signals (e.g. velocity, P-to-S velocity ratio, main frequency, and main frequency amplitude) are a result of the internal micro-structure change of the GHBS samples. Therefore, the acoustic responses during creeping could be used to explain the deformation mechanisms, as well as to monitor and characterize failure processes of the GHBS during NGH exploitation.

From the failure processes monitoring and characterization perspectives, the acoustic velocity, compression-to-shear velocity ratio and Poisson’s ratio evolve continuously before the sample is destroyed. The sudden drop of the acoustic velocity or compression-to-shear velocity ratio appears almost simultaneously with the occurrence of the accelerated creep stage. Therefore, they can be used as indicators for the failure of GHBS, while they cannot be used as early warnings for the coming creep failure. On the other hand, both the main frequency and its amplitude exhibit abnormal changes under the condition when the axial load approaches the long-term strength of the GHBS or when the accelerated creep appears. Therefore, the fluctuations of the main frequency and its amplitude could be used as indicators for both early warnings and failure characterization for GHBS. Typical changing modes for the main amplitude and its amplitude obtained from sections “Main frequency” and “Main frequency amplitude” are summarized in Table 2. However, some of these indicators remain constant (marked with ‘*NA*’ in Table 1) under some certain conditions and are unable to predict or characterize creep failure. This might be attributed that some acoustic signals are not sensitive to some certain failure modes. Therefore, it is difficult to predict the creep failure of GHBS by solely deploying only one of these abnormal changes. The combination of the main frequency and main frequency amplitude of both S-wave and P-wave is essential for advanced forecast of creep failure of GHBS.

**Table 2:** Main frequency and its amplitude abnormal fluctuation modes prior to the emergence of accelerated creep for sandy HBS.

	Main frequency		Main frequency amplitude	
	P-wave	S-wave	P-wave	S-wave
*S*_h_ = 30%, *P*_c_ = 1.0 MPa	*NA*	*NA*	Plummet	Plummet
*S*_h_ = 30%, *P*_c_ = 2.0 MPa	*NA*	Sharp increase	Plummet	/
*S*_h_ = 30%, *P*_c_ = 3.0 MPa	Plummet	Plummet	/	/
*S*_h_ = 20%, *P*_c_ = 1.0 MPa	*NA*	*NA*	Plummet	*NA*
*S*_h_ = 40%, *P*_c_ = 1.0 MPa	Sharp increase	*NA*	*/*	*NA*

Note: ‘*NA*’ indicates that this parameter remains unchanged at when the axial load approaches the long-term strength. Two changing modes are observed in this study for main frequency, namely plummet and sharp increase. ‘/’ indicates that this parameter is invalid for advanced forecast of the coming creep failure. The main frequency amplitude decreases in all cases.

As was stated above, main frequency is the first indicator for advanced forecast of the coming creep failure. The secondary indicator (namely the main frequency amplitude) is valid only if the main frequency remains unchanged. Those invalid fluctuations of the main frequency amplitude are marked with “/” in Table 2. It could be seen that the main frequency shows two fluctuation modes of either sudden plummet or sharp increase, whereas the main frequency amplitude plummets in all cases.

From the perspective of creep failure mechanisms, only the transmitted signals could be detected in the test, whereas the others are either scattered or reflected^62^. The main frequency amplitude represents the strength of acoustic energy^63^. The increase in the main frequency amplitude implies the enhancement of acoustic conductivity and homogeneity of the sample, whereas the decrease in the main frequency amplitude indicates damage and structural disorder inside the sample^64^. Therefore, the fluctuations for the main frequency amplitude might indicate the amount of fractures or the propagation scales of fractures within the HBS. Before the axial load reaches the long-term strength, the main frequency amplitude for both P-wave and S-wave remain relatively stable, whereas the changing trends for P-velocity and S-wave velocity remain parallel (see Fig. 5). This indicates that the HBS remains undamaged during this stage, however, micro-structural adjustments such as particle rotation and movement occur dynamically.

Regarding the influence of fracture orientations on acoustic responses within marine hydrate reservoirs, Lee and Collett^65^ proved that hydrate saturation estimated from P-wave velocities assuming vertical fractures agree well with those from the pressure cores. Afterwards, Wang et al.^66^ anticipated that the P-wave is more sensitive to fractures that are parallel or sub-parallel to the normal stress, whereas the S-wave is more sensitive to fractures that are perpendicular or sub- perpendicular to the normal stress. This gives us significant implications that whether the main frequency fluctuations occur on the P-wave or S-wave can be used as an indicator to identify the failure orientations. However, further careful work and emerging technics are needed to quantitatively evaluate the relationships between fracture orientations and main frequency fluctuations for GHBS.

All in all, the creep failure mechanisms of GHBS are completely different from the homogeneous single-phase geomaterials. The combination of different acoustic parameters provides an effective way for qualitative early warnings, as well as multi-dimensional characterization for creep failure of GHBS. However, we believe that the qualitative characterization and prediction of the creep failure processes for GHBS are far to be well-solved. A more advanced testing method that coupling the micro-tomography techniques is needed to disclose these mysteries.

## Conclusions and suggestions

1. In this study, creep behaviors of the sandy GHBS and their acoustic responses are jointly analyzed through multi-step loading creep test. It is proved that the GHBS undergoes three different deformation stages of deceleration creep, stable creep, and accelerated creep during long-term loading. Under the same effective confining pressure and the same hydrate saturation, the long-term strength derived from creep tests is proved to be 0.45–0.60 times of the shear strength derived from triaxial shearing. Geoengineering risk prediction and evaluation methods that ignore the influence of the creep might underestimate the scale and intensity of possible geoengineering risks during long-term NGH exploitation.
2. It is proved that the acoustic signals could give insightful information to characterize the creep failure processes of the GHBS. The velocity increases gently at the early stages of creep and then decreases continuously at the middle-later stage. The P-wave velocity, S-wave velocity, and the P-to-S wave velocity ratio plummet suddenly at the moment when accelerated creep stage appears. Therefore, the plummet of acoustic velocity and P-to-S wave velocity ratio can be used as prominent identifications for creep failure for GHBS, whereas Poisson’s ratio fails to indicate creep failure of the GHBS in most cases.
3. The abnormal fluctuation of main frequency and its amplitude usually appear prior to the occurrence of the accelerated creep stage. On one hand, this highlights the reasonability to detect internal structure changes for GHBS via acoustic signals, especially for those micro-changes that are unable to be detected by the strain gauges. Most importantly, it enlightens us on the possibility of advanced forecast of creep failure for GHBS. However, the sensitivity of main frequencies of P-wave and S-wave is different for different micro-structure failure modes. A combination of abnormal fluctuations of main frequency and its amplitude is essential for advanced forecast of creep failure.
4. This study recognized the main acoustic indicators for both real-time characterization and early-warnings of possible creep failure during long-term NGH exploitation, and proved the possibility of in-situ non-destructive advanced forecast and detection of creep failure for GHBS via the application of geophysical techniques. One of the main challenges in characterizing the creep failure of GHBS is to characterize the fracture propagation direction and its geometric shapes. A more advanced testing method that coupling the micro-tomography techniques is needed to determine these uncertainties.

## Data Availability

The datasets used and/or analyzed during the current study available from the corresponding author on reasonable request.
